# Mid‐term Clinical Outcomes of “Light Bulb” Core Decompression with Arthroscopic Assistance in Peri‐collapse Osteonecrosis of the Femoral Head: A Retrospective Comparative Study

**DOI:** 10.1111/os.14058

**Published:** 2024-05-07

**Authors:** Jibin Yang, Pengpeng Sun, Ziming Liu, Yuwan Li, Jun Zhang, Yi Liu, Gang Zou

**Affiliations:** ^1^ Department of Orthopedic Surgery Affiliated Hospital of Zunyi Medical University Zunyi China; ^2^ Beijing Key Laboratory of Sports Injuries, Department of Sports Medicine Institute of Sports Medicine of Peking University, Peking University Third Hospital Beijing China; ^3^ Department of Orthopedics The First Affiliated Hospital of Chongqing Medical University, Orthopedic Laboratory of Chongqing Medical University Chongqing China

**Keywords:** Core decompression, Hip arthroscopy, Light bulb, Necrosis of the femoral head, Peri‐collapse

## Abstract

**Objective:**

Nontraumatic osteonecrosis of the femoral head (ONFH) is commonly encountered in orthopedics. Without early clinical intervention, most patients with peri‐collapse of the ONFH will develop femoral head necrosis and eventually require hip replacement surgery. The aim of this study is to evaluate clinical outcomes in patients with ONFH who underwent “light bulb” core decompression (CD) with arthroscopic assistance and to compare them with the outcomes of those treated with traditional procedures.

**Methods:**

A retrospective review of patients with Stage II and IIIA (Peri‐collapse) radiographic findings based on the Association Research Circulation Osseous (ARCO) stage for ONFH who underwent “light bulb” CD with or without arthroscopic assistance by a single‐surgeon team between March 2014 and December 2018 was performed. All patients were followed up for a minimum of 2 years. The visual analogue scale (VAS) pain score, Harris hip score (HHS), and radiological imaging were evaluated. The categorical parameters were analyzed by chi‐square test and the continuous variables conforming to a normal distribution were analyzed by Student's *t*‐test.

**Results:**

The study included a total of 39 patients (18 and 21 patients in the with and without arthroscopic assistance groups, respectively), with a mean age of 40.3 years and a mean follow‐up of 22.2 months. Overall, there was a better VAS score in the arthroscopic assistance group than in the control group (*p* < 0.05), There was a significant difference in HHS (80.1 ± 9.2 *vs* 75.1 ± 12.7) at the last follow‐up (*p* < 0.05). The rate of good and excellent outcomes was 94%. Similarly, there was no significant difference in the total rate of complications or conversion to THA.

**Conclusion:**

With arthroscopic assistance, “light bulb” CD could be achieved via hip arthroscopy with less trauma, and it offered the opportunity for more precise evaluation and monitoring for therapy and yielded better VAS scores after surgery and better hip function outcomes at the last follow‐up.

## Graphical abstract

Hip arthroscopy is a very effective assistive technique in “light bulb” core decompression for the treatment of pre‐collapse nontraumatic osteonecrosis of the femoral head.

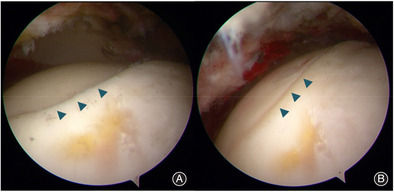

## Introduction

Nontraumatic osteonecrosis of the femoral head (ONFH) is commonly encountered in orthopedics and occurs in young and middle‐aged people between 20 and 50 years old.^1^, ^2^ Without early clinical intervention, most patients with peri‐collapse period of ONFH will develop femoral head necrosis and secondary hip osteoarthritis within 5 years and eventually require hip replacement surgery.^3^, ^4^ In the peri‐collapse period, when the cartilage collapse is less than 2 mm, femoral head preservation treatments are performed, including core decompression (CD), nonvascularized bone grafting, vascularized bone grafting and proximal femoral osteotomies.^2^ The aims of these procedures are to reduce intraosseous hypertension, restore blood supply, and relieve pain.^5^

There are three major methods used for CD: the traditional procedure via the lateral side of the greater trochanter of the femur, the trapdoor procedure via the cartilage of the femoral head, and the “light bulb” procedure through a window at the femoral head–neck junction.^6^ Traditional CD, which occurs via the lateral side of the greater trochanter of the femur, has a varied success rate, and 36% of patients need THR 30 months postoperatively.^7^ The trapdoor procedure requires dislocation of the hip and an open trapdoor of cartilage, which is usually used for treating Ficat and Arlet stage III osteonecrosis.^8^

The “light bulb” technique is used for early ONFH treatment, with a femoral head survival rate of up to 81% in the initial study and more than 90% in later studies.^9^ The “light bulb” procedure via the direct anterior approach (DAA) is less invasive, results in moderate blood loss, and requires less than 1 h of surgery time. Moreover, when surgery fails, it does not increase the operative difficulty of THA.^10^ Recently, Wang *et al*. reported that the “light bulb” procedure via a DAA offers significantly better results than traditional CD.^9^

Arthroscopy and navigation have been used to support CD to improve the first‐pass accuracy and reduce intraoperative radiation.^11^ Several studies have reported using arthroscopic assistance to treat ONFH, and hip arthroscopy could be used for classifying peri‐collapse subchondral insufficiency fractures and aiding treatment.^12^, ^13^ Arthroscopic assistance provides both diagnostic and therapeutic aid, and hip arthroscopy could enable precise classification of the cartilage injury and treatment of concomitant intra‐articular pathology, as well as provide direct visibility of the hip during treatment.

Guadilla *et al*. used a hip arthroscopy‐assisted “light bulb” technique for CD and bone graft implants and found that arthroscopic management of ONFH is viable and offers significant advantages with additional benefits.^14^. The effect of ceramic rod osteogenesis is good. Many patients can effectively treat early osteonecrosis of the femoral head by core decompression and implantation of the ceramic rod, but this kind of operation through the incision of the lateral wall of the femur may not be able to remove the focus tissue better. At the same time, there will also be a larger surgical incision, the arthroscopy‐assisted “light bulb” technique can more intuitively observe the location of the focus of the femoral head. At the same time, in the process of implanting ceramic rods, we can clearly observe whether the implantation depth can support the femoral head intact. In this study, we retrospectively examined cases of early stage nontraumatic ONFH that were treated with the arthroscopy‐assisted “light bulb” technique and compared this procedure with traditional CD.

The purpose of this study was: (i) to investigate the outcome of “light bulb” CD with arthroscopic assistance; and (ii) to explore the advantages of “light bulb” CD with arthroscopic assistance. We hypothesize that based on this retrospective study, the arthroscopically assisted “light bulb” technique is able to treat ONFH by achieving accurate and minimally invasive decompression.

## Methods

The inclusion criteria were as follows: (i) patients with radiographic findings of Stage II and IIIA (Peri‐collapse) based on the Association Research Circulation Osseous (ARCO) stage for OFNH; (ii) arthroscopic or non‐arthroscopic “light bulb” technique for the treatment of OFNH; and (iii) follow‐up of a minimum of 2 years. The exclusion criteria were (i) greater severity based on the ARCO stage; (ii) dysplastic hips, and (iii) complications of consumptive disease.

### General Characteristics of Participants

This study was approved by the Ethics Committee of our institution (2021060301). Written informed consent was obtained from all participants.

### Patients

Patients who underwent hip‐preserving operations between March 2013 and December 2018 in our institution were identified. Patients diagnosed with nontraumatic ONFH were subsequently identified. Patients were divided into two groups according to the type of surgery received: the arthroscopic assistance group, which underwent the hip arthroscopy‐assisted “light bulb” technique for CD and bone graft implantation, and the control group, which received CD and bone graft implantation via the lateral side of the greater trochanter of the femur.

### Surgical Procedures

#### Establishment of Arthroscopic Approach

**FIGURE 1:**
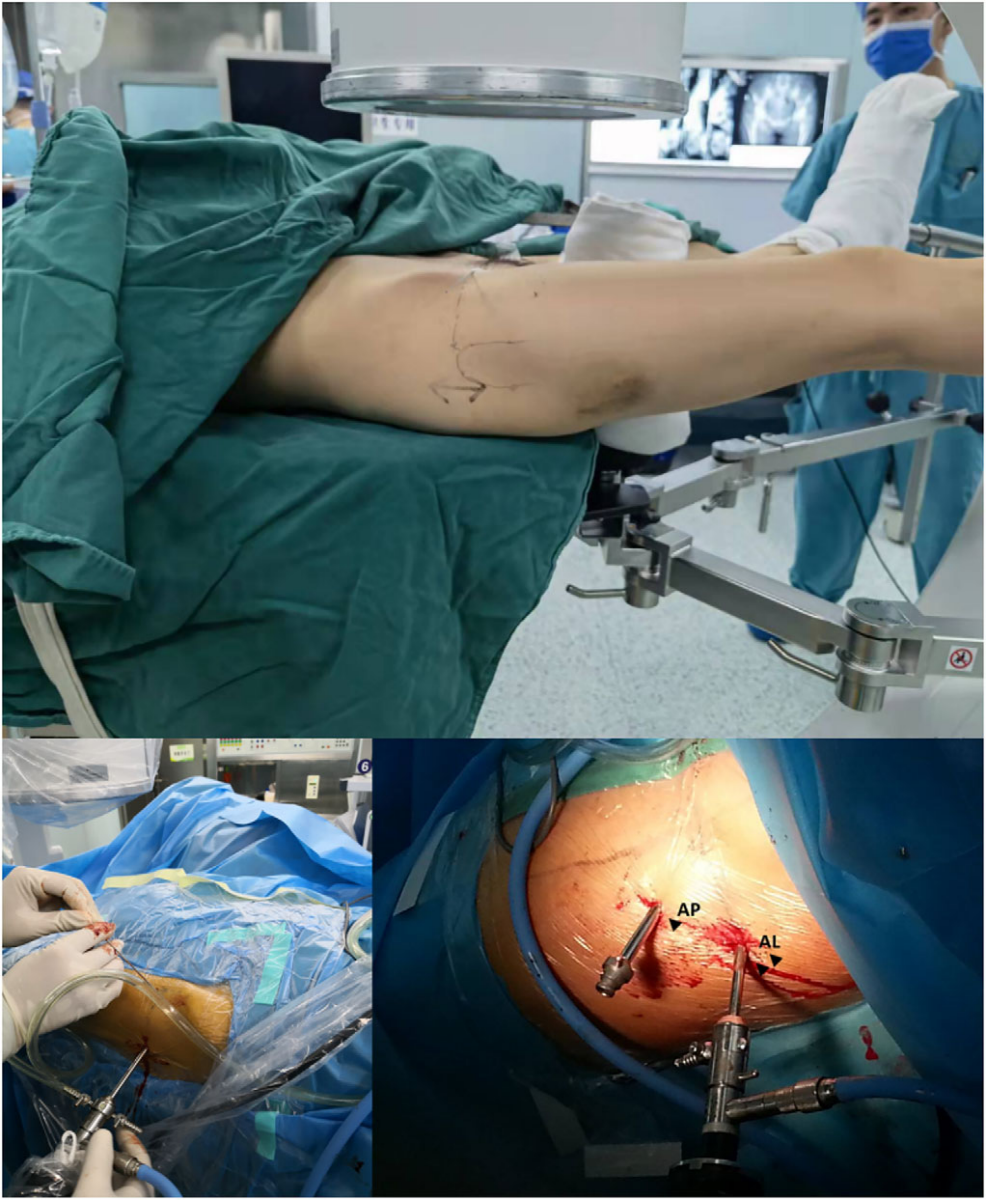
Patient position and diagnostic arthroscopy.

**FIGURE 2:**
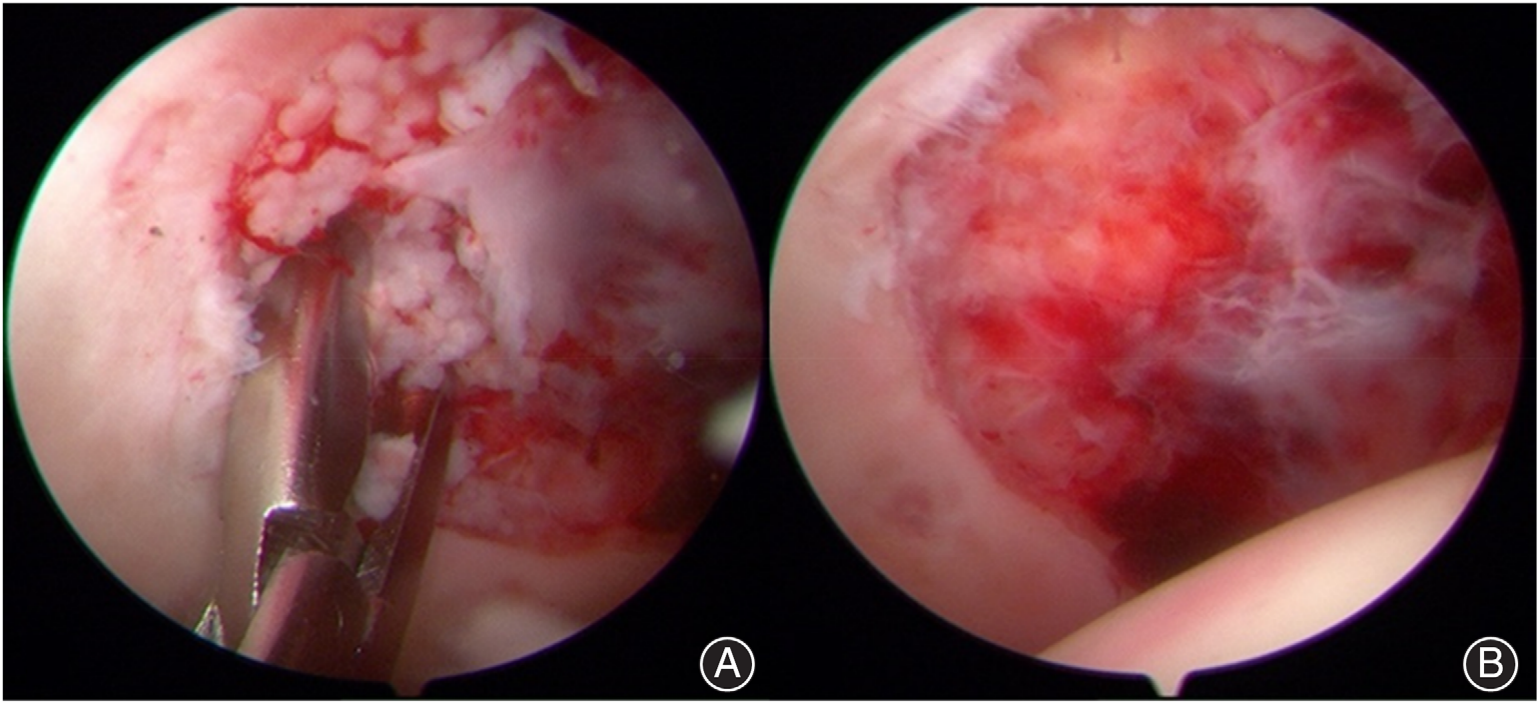
Intraoperative arthroscopic image demonstrating intra‐articular pathology, including loose bodies, pincer lesions, cam lesions, synovitis, labral tears, and chondral defects.

After general anesthesia, for patients in the arthroscopic assistance group, the patient was placed on an orthopedic traction bed with a thicker perineum column, and the feet were protected by cotton pads and placed in the boots of the traction bed. Under the guidance of the C‐arm image intensifier, a standard anterolateral approach for hip arthroscopy was established. The remaining entrances (anterior, midanterior, and posterolateral) were established under arthroscopic monitoring, and the above four entrances were used according to the location of the necrosis. An arthroscopic examination of the hip joint was performed, including assessment of the femoral head for the presence of collapse, assessment of the articular cartilage overlying the acetabulum, probing of the articular cartilage to identify any softening or chondral flaps, and inspection of the condition of the chondrolabral junction. Synovectomy was also performed if significant synovitis was observed (Figures 1 and 2).

#### Opening a Cortical Window

**FIGURE 3:**
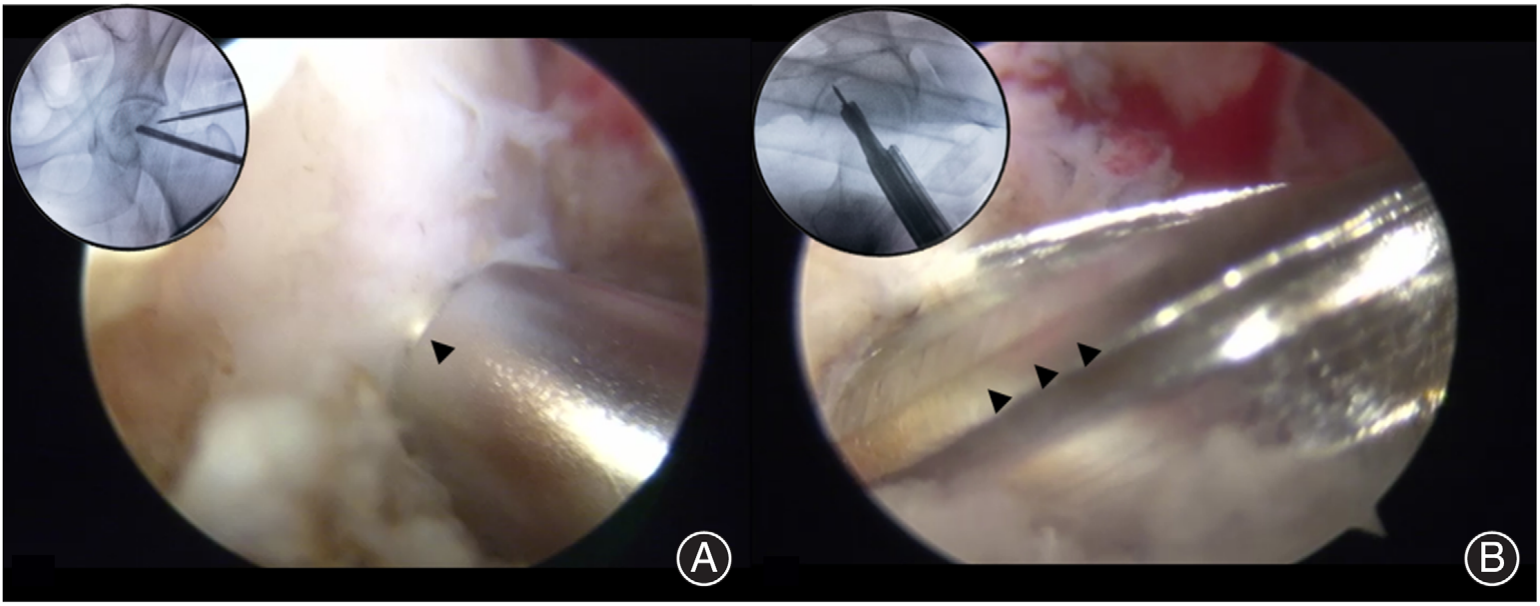
Opening a cortical window at the level of the head–neck junction under monitoring by hip arthroscopy.

Patients at stages II and III, with moderate or severe cystic and sclerotic changes, required full debridement of the necrotic tissue and subsequent bone grafting. To achieve full debridement, the “light bulb” approach was used. After opening a cortical window at the level of the head–neck junction, the necrotic area was approached using a Kirschner pin, and the proper trajectory for the trephine drill was confirmed by fluoroscopy. The 11‐mm cannula was adjusted, and a 10‐mm trephine drill was advanced through the cortical window into the necrotic niche. Core healthy bone was recovered for subsequent grafting, while the necrotic bone was removed with either burrs or curettes (Figure 3).

#### Focus Debridement and Bone Grafting

**FIGURE 4:**
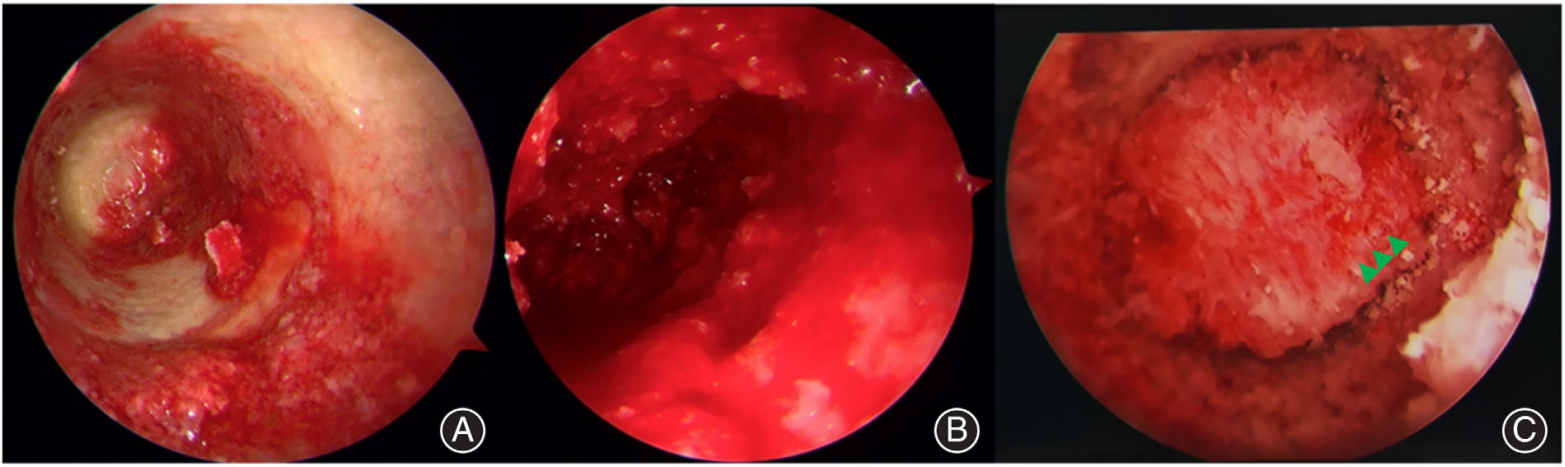
The necrotic bone is removed and refilled with bone graft.

**FIGURE 5:**
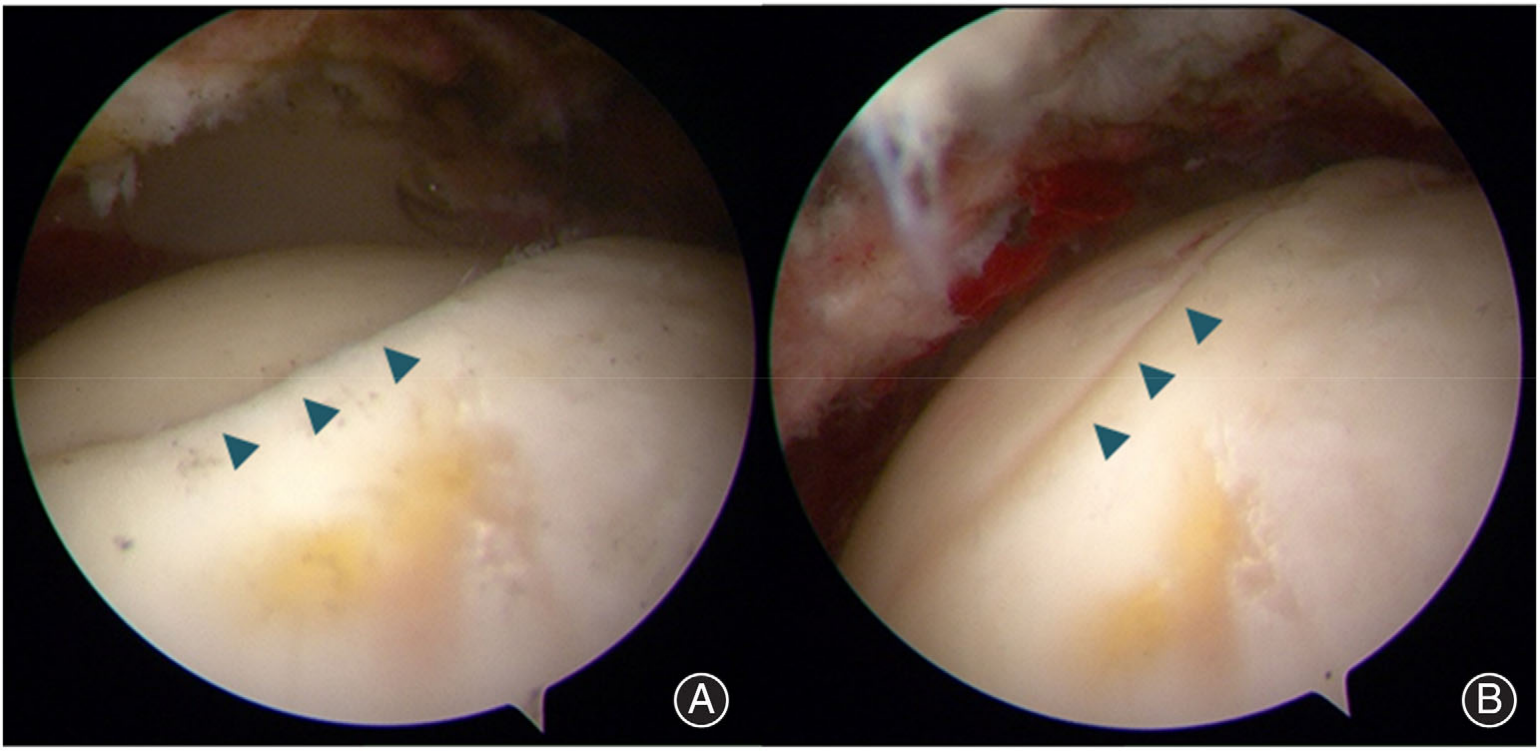
The Peri‐collapsed femoral head was restored under monitoring by hip arthroscopy.

The bone graft—ideally ipsilateral iliac cancellous bone combined with biological ceramic—was introduced through the trephine drill track, as described below. The autogenous iliac cancellous bone particles were mixed with bioceramic particles to fill the necrotic area of the femoral head, and the bone tunnel was sealed with an iliac bone column (Figures 4 and 5).

After completion of the procedure, the arthroscope and tools were removed, and the portals were closed in a routine manner.

#### Traditional Incision of Core Decompression and Ceramic Rod Transplantation

Patients in the control group were placed under spinal anesthesia in the supine position on an orthopedic traction bed. Under monitoring with a C arm, a Kirschner wire (2.5 mm) was placed in the center of the lesion in the femoral head, just proximal to the level of the lesser trochanter. A small incision was made where the Kirschner wire entered the skin. After incising the skin, the reamer (10 mm) was slowly introduced into the necrotic area. Through the bone tunnel, with the help of a curette, the necrotic bone could better be cleaned up. Autogenous iliac cancellous bone particles were mixed with bioceramic particles to fill the necrotic area of the femoral head, and the bone tunnel was sealed with a bioceramic rod.

### Postoperative Rehabilitation and Follow‐up

Patients were instructed to ambulate using crutches with a flat‐foot gait for protected weight bearing for 6–8 months. Postoperative X‐rays were obtained at the 8‐week visit to evaluate the condition of the femoral head. The primary outcomes were conversion to THA, the visual analogue scale (VAS) pain score and the Harris hip score (HHS). In addition, the time intervals for both procedures were calculated. Secondary data points included patient demographics (age, sex, body mass index [BMI]), symptomatic interval duration before surgery, and arthroscopic findings. Finally, complications were evaluated, including both major complications, such as subtrochanteric femur fracture, violation of articular cartilage, extra‐articular fluid extravasation, hip dislocation, thromboembolism, and joint sepsis; and minor complications, including adhesions, neurapraxia, broken instrumentation, superficial wound infection, and heterotopic ossification.

### Statistical Analysis

Statistical software IBM SPSS 25 (IBM, Armonk, NY, USA) was used for statistical analyses. The HHSs and VAS scores preoperatively, 3 days postoperatively and at the last follow‐up were compared. For data with a normal distribution, we used an unpaired sample *t*‐test for the comparison test. For data with a nonnormal distribution or uneven variance, we used the Mann–Whitney *U* test. Differences with *p* values < 0.05 were significant.

## Results

### Demographic Data

A total of 39 patients were identified; 18 patients (16 males, two females) were included in the arthroscopic assistance group, and 21 patients (18 males, three females) were included in the control group. There were no significant differences in age, BMI or preoperative ARCO staging (Table 1).

**TABLE 1:** Patient demographics.

Clinical data	Arthroscopically assisted group (*n* = 18)	Control group (*n* = 21)	*t*	*p*‐value
Age, years ± SD	39.7 ± 8.5	40.8 ± 10.2	0.822	0.416
BMI, BMI ± SD	22.8 ± 2.9	21.6 ± 2.9	−0.239	0.813
Gender				
Male	16	18		
Female	2	3		
Pre‐op ARCO staging				
II	13	17		
III	5	4		
Follow‐up time (months)	38.3 ± 18.9 (24–90)	34.6 ± 8.2 (24–55)	−0.518	0.607
HHS at preoperative	59.8 ± 7.7	57.5 ± 5.8	0.075	0.941
HHS at last follow‐up	80.1 ± 9.2	75.1 ± 12.7	−1.627	0.112
VAS at preoperative	5.7 ± 0.9	4.4 ± 1.3	−3.040	0.004
VAS at postoperative (3 day)	2.0 ± 0.3	2.8 ± 0.5	6.358	0.000
VAS at last follow‐up	1.2 ± 2.2	2.2 ± 2.8	1.107	0.276
Conversion to THA *n* (%)	2 (11.1%)	6 (28.6%)		
Time from CD to THA (mo)	26.0 ± 2.0	41.3 ± 8.7		

Abbreviations: BMI, body mass index; CD, core decompression; HHS, Harris hip score; THA, total hip arthroplasty; VAS, visual analogue scale.

**FIGURE 6:**
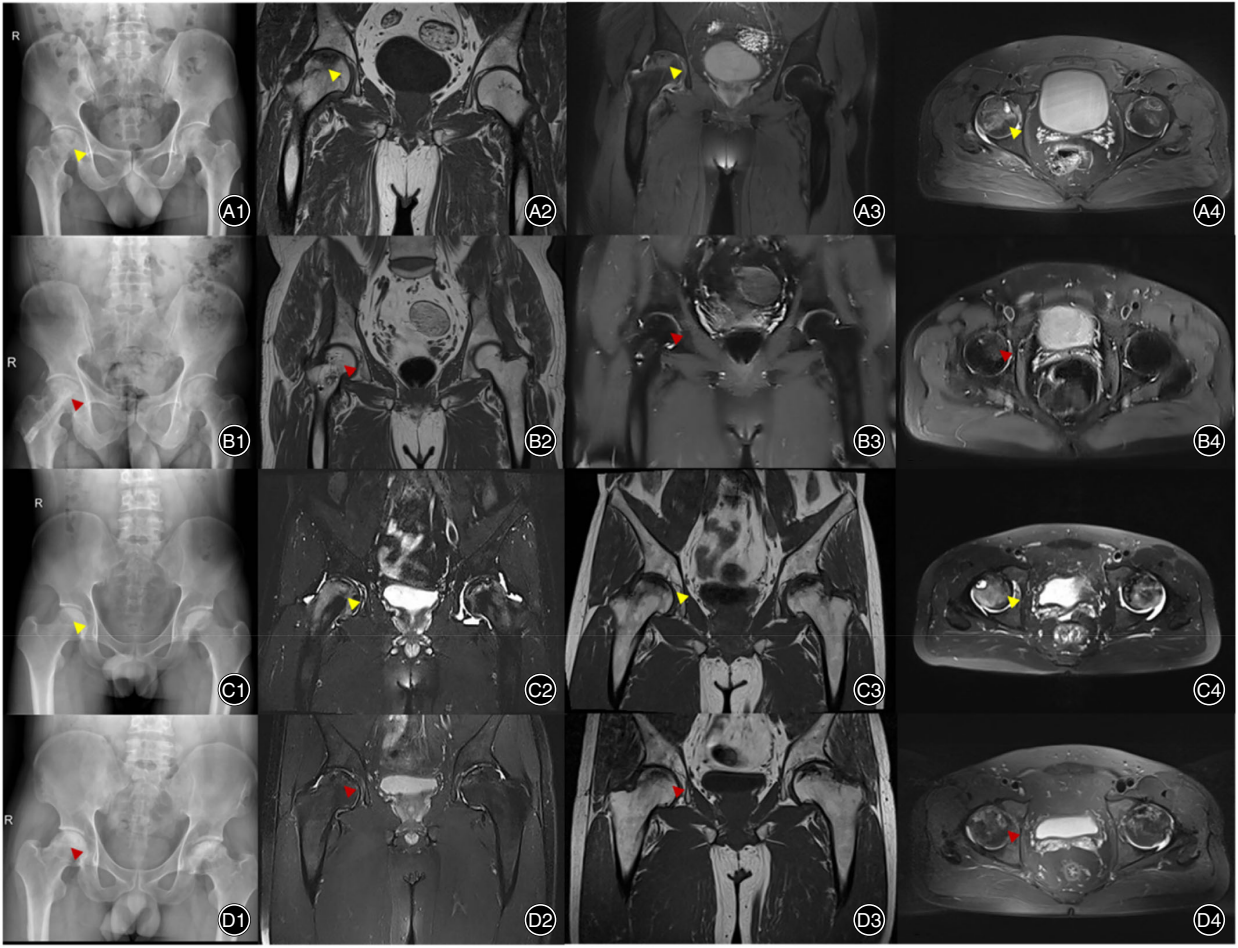
Preoperative and last follow‐up magnetic resonance imaging (MRI) images for patients who received arthroscopically assisted “light bulb” core decompression (CD) and bone graft implantation.

In each group, there was a significant difference between the preoperative and postoperative VAS scores and HHS. Between the two groups, there was a significant difference in VAS scores (2.0 ± 0.3 *vs* 2.8 ± 0.5) (*p* < 0.05) 3 days postoperatively but no difference at the last follow‐up. There was a significant difference in HHS (80.1 ± 9.2 *vs* 75.1 ± 12.7) at the last follow‐up (*p* < 0.05). Magnetic resonance imaging (MRI) showed that in both groups, most of the patients' femoral heads were successfully reconstructed after the operation (Figure 6).

The postoperative follow‐up X‐rays showed that in two patients (11.1%) in the arthroscopic assistance group and six patients (28.6%) in the control group, the femoral head had collapsed and that the treatment had failed and required total hip replacement.

The most common concomitant pathologies addressed at the time of arthroscopy were synovectomy and microfracture (Table 2) There were no major complications (0%), including subtrochanteric fracture and violation of the articular cartilage, or minor complications.

**TABLE 2:** Arthroscopic findings and procedures.

Condition and management of patient's joint	*n*
Arthroscopy findings	
Cartilage wear	15
Chondral defect	2
Cartilage collapse	4
Synovitis	12
Labral tear	4
Cam lesion	2
Chondroma	1
Arthroscopy procedure	
Microfracture	14
Reduction of cartilage collapse	4
Synovectomy	12
Labral debridement	3
Femoral osteoplasty	1
Labral repair	1

## Discussion

In this study, we retrospectively assessed functional recovery, clinical outcomes, radiographic outcomes, and survival in two groups of patients who were diagnosed with early stage nontraumatic ONFH and underwent arthroscopically assisted “light bulb” procedures or traditional CD surgery. The most important feature of the described technique is the ability to precisely evaluate and monitor the restoration of femoral head collapse with the assistance of hip arthroscopy. Diagnostic arthroscopy can also be used to perform a full scan of the hip, and borderline developmental dysplasia of the hip (BDDH), femoroacetabular impingement (FAI) and labral tears were evaluated under hip arthroscopy. The main finding of this study is that arthroscopically assisted hip examination and CD with bone grafting provided clinical benefit, especially in terms of postoperative VAS score and hip function at the last follow‐up.

### Comparison of Arthroscopy‐assisted “Light Bulb” Decompression and Traditional Core Incision Decompression

Surgical intervention is an effective treatment for peri‐collapse ONFH that reduces hip pain, improves hip function and delays the need for THA.^2^, ^4^ The aims of these procedures, including CD and adjunctive bone grafting, were to remove all necrotic tissues and implant new bone grafts to restore the subchondral bone of the femoral head.^15^ Several previous studies have shown that “light bulb” CD is the most effective procedure for treating peri‐collapse ONFH, especially in the early stage.^2^, ^9^, ^16^

“Light bulb” CD was first described by Rosenwasser *et al*. and was report to have an 87% femoral head survival rate in a mean follow‐up period of 12 years.^16^ Subsequent studies showed better results, with a femoral head survival rate as high as 90%.^9^, ^17^ Our results support these findings; there was a near 90% survival rate of the femoral head after “light bulb” CD procedures and better hip function at the last follow‐up compared with traditional CD. These results may be because “light bulb” CD allowed full debridement of necrotic bone with a direct approach via the femoral head–neck junction, and this approach is also easily used for implanting bone grafts, which are vital for reconstructing the structure of the femoral head and subchondral bone.

In our study, the assistance of hip arthroscopy brought many clinical benefits for surgery and recovery. First, arthroscopy could provide a direct view of osteochondral lesions and fractures of the femoral head and enabled monitoring of the restoration of femoral head cartilage. A previous study showed that hip arthroscopy enables the classification and treatment of peri‐collapse subchondral insufficiency fractures of the femoral head.^13^ After diagnosing and classifying cartilage collapse, arthroscopy is used to monitor complete removal of necrotic bone tissue and implanted bone and directly view the femoral head cartilage restoration.

“Light bulb” CD procedures were approached via the Watson–Jones or Smith–Petersen approach,^6^, ^16^ while Wang *et al*. reported using the DAA approach.^9^ A previous study also reported an arthroscopically assisted “light bulb” procedure;^14^ however, they used the Watson–Jones approach to complete “light bulb” CD and bone graft implantation after arthroscopic inspection.^6^, ^14^ In our study, we finished the whole operation under arthroscopic assistance. “Light bulb” CD and bone graft implantation were performed via the AP or AL hip arthroscopic approaches. Special instruments were used to help access the level of the head–neck junction, which reduced the incision and tissue damage. In our study, we also found that the VAS scores were better in the arthroscopic assistance group than in the control group 3 days postoperatively. Additionally, arthroscopically assisted retrograde drilling of the humeral head with a guiding device was reported to show that arthroscopy is an efficient tool.^18^

In our study, full arthroscopic inspection of the hip was performed on all patients in the arthroscopic assistance group. Our most common findings were cartilage wear and synovitis, and the subsequent procedures were microfracture and synovectomy. Some studies have also shown that arthroscopy could assist in treating pathologies in the hip during surgery for CD.^12^, ^13^ These lesions in the hip could appear before or after ONFH, and hip arthroscopic surgery is one of the best therapies.^19^ No studies have shown whether these hip pathologies exacerbate the progression of femoral head necrosis or affect the outcome of surgery, but they do induce degeneration of the hip and elicit pain.

### Diverse Findings and Influences

Although there is controversy surrounding hip arthroscopic surgery in the setting of osteoarthritis, McCarthy *et al*.^20^ showed that debridement could relieve symptoms in patients with early hip disease. Eventually, therapy for hip lesions offers clinical benefits for patients, as evidenced by the arthroscopic assistance group achieving better hip function than the control group at the last follow‐up.

There are also some risks of hip arthroscopy, the complications of hip arthroscopy are mostly related to hip distraction and portal placement during the procedure. Others like septic arthritis, neuropraxia, hemorrhage, bursitis, instrument breakage, chondral and labral damage and fluid extravasations were the complications of hip arthroscopy. So to help patients achieve the most benefits of this procedure, the surgeon should get training before to master skills and also strictly control the symptoms.

### Limitation and Prospect

There were several limitations in this study. First, with the rapid development of arthroscopic technology, more and more diseases will be treated by minimally invasive surgery. Arthroscopic surgery can solve these problems and accelerate the rehabilitation process of patients. However, the learning curve of arthroscopy is long, especially hip arthroscopy, which has not been carried out in most areas at present. Therefore, there were a limited number of cases in this study, as is common in retrospective comparative studies, and the lack of randomization and blinding is an obvious limitation of this study. The patients and the surgeons who performed the operation were not informed before the surgery to reduce potential bias. Second, the control group underwent CD and bone graft implantation via the lateral side of the greater trochanter of the femur. In future studies, we will compare “light bulb” CD with and without arthroscopic assistance. Third, most of our patients had ARCO stage II disease, which is an early stage of ONFH. The clinical results of arthroscopically assisted “light bulb” CD and bone grafting for severe ONFH are still not clear, and the indications for this procedure are still limited. Finally, the risk of arthroscopy and the benefits were only discussed in this small sample size, in future large sample size with multicenter random clinical trials should be provided.

## Conclusion

With arthroscopic assistance, “light bulb” CD could be accomplished via hip arthroscopy with less trauma, and it offered more precise evaluation and monitoring for therapy and yielded better VAS scores after surgery and better hip function outcomes at the last follow‐up.

## Author Contributions

Conception and design: JY and PS. Administrative support: GZ. Provision of study materials: ZL and YL. Collection and assembly of data: JZ and PS. Data analysis and interpretation: JY and YL. Manuscript writing: JY. All authors contributed to the article and approved the submitted version.

## Conflict of Interest Statement

The authors declare that they have no competing interests.

## Ethics Statement

The data sources are approved by the Ethics Committee of the affiliated Hospital of Zunyi Medical University and meet the requirements of ethical norms.

## Supporting information


**Figure S1.** Shows the treatment of osteonecrosis of the femoral head: osteonecrosis of the femoral head (A), core decompression and ceramic rod support (B), hip arthroplasty (C).
