# Supplementary material for: Mid‐term Clinical Outcomes of “Light Bulb” Core Decompression with Arthroscopic Assistance in Peri‐collapse Osteonecrosis of the Femoral Head: A Retrospective Comparative Study
Source: Orthop Surg. 2024 May 7;16(6):1399–406. doi: 10.1111/os.14058 (PMC11144504; doi:10.1111/os.14058)
Supplement: Supplementary file 1 — Figure S1. Shows the treatment of osteonecrosis of the femoral head: osteonecrosis of the femoral head (A), core decompression and ceramic rod support (B), hip arthroplasty (C). [file OS-16-1399-s001.docx]

A


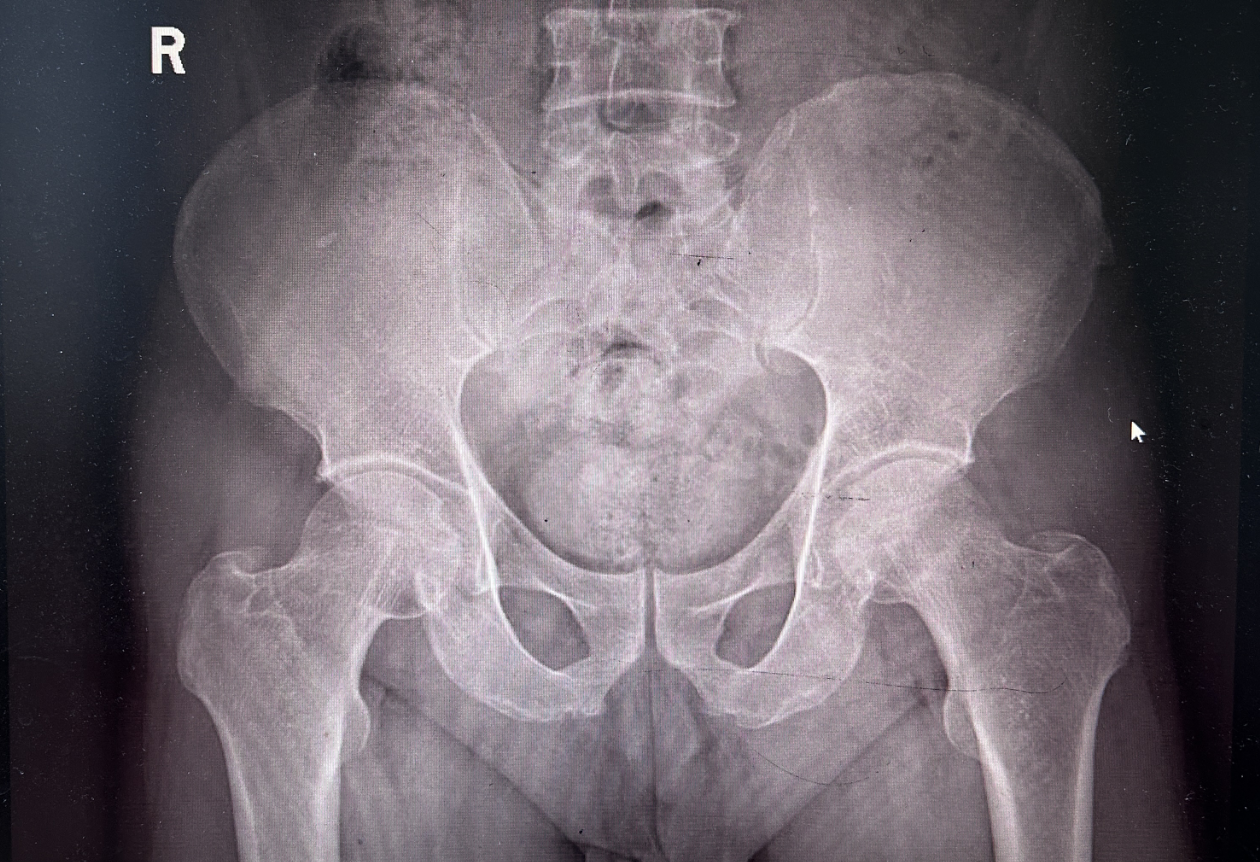


B


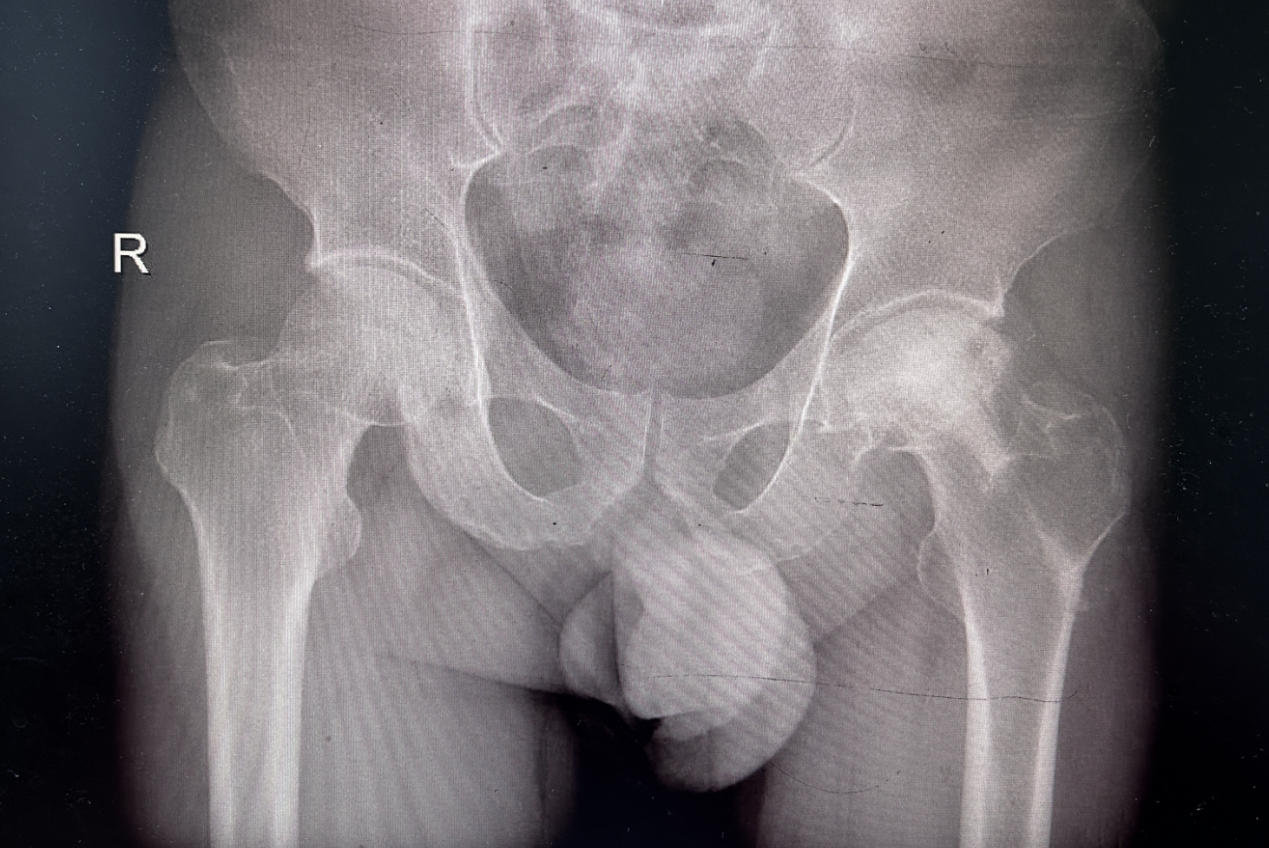


**C**


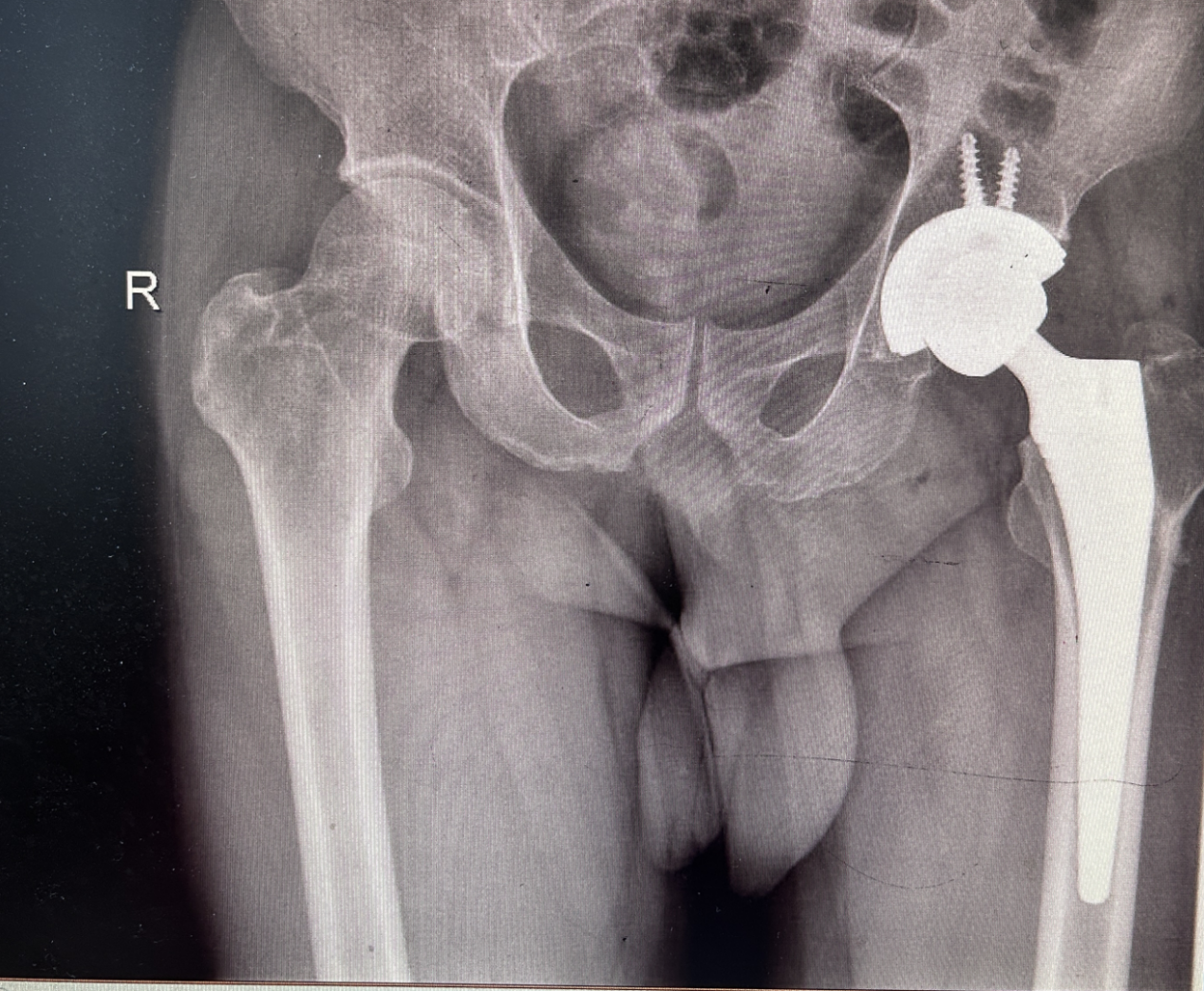


**Figure 1 shows the treatment of osteonecrosis of the femoral head: osteonecrosis of the femoral head (A), core decompression and ceramic rod support (B), hip arthroplasty (C)**
